# Supplementary material for: SARS-CoV-2 Infection in Pregnancy: Clues and Proof of Adverse Outcomes
Source: Int J Environ Res Public Health. 2023 Feb 1;20(3):2616. doi: 10.3390/ijerph20032616 (PMC9915124; doi:10.3390/ijerph20032616)
Supplement: Supplementary file 1 [file ijerph-20-02616-s001.zip › ijerph-2121638-supplementary.pdf]

Table S1. Comparison of SARS-CoV-2 positive with SARS-CoV-2 negative pregnant women and their children.

	SARS-CoV-2 positive pregnant women (n=68)	SARS-CoV-2 uninfected pregnant women (n=136)	*p-value*	Odds ratio	95% C.I.
Preterm birth (%)	12.31	9.56	0.55	1.33	0.52 - 3.38
Cesarean section (%)	38.24	38.97	0.92	0.97	0.53 - 1.76
**Chronic diseases (%)**					
total	14.71	17.91	0.57	0.79	0.35 - 1.76
Hypertension	1.47	0.00	1.00	6.07	0.24 - 150.92
Diabetes	4.41	0.74	0.11	6.23	0.64 - 61.07
Hypothyroidism	7.35	10.29	0.50	0.69	0.24 - 2.01
Obesity	2.94	0.00	0.26	10.26	0.49 - 216.83
**Pregnancy-associated complications (%)**					
total	44.12	18.38	*0.0001*	3.51	1.84 - 6.69
Hypertension	4.41	2.21	0.40	2.05	0.40 - 10.42
Diabetes	17.65	5.88	*0.008*	3.43	1.33 - 8.85
Hypothyroidism	7.35	0.00	*0.017*	23.65	1.29 - 434.22
Cholestasis	0.00	2.21	1.00	0.28	0.01 - 5.47
Preeclampsia	2.94	0.00	0.26	10.26	0.49 - 216.83
Intrauterine growth restriction	10.29	0.00	*0.002*	33.29	1.87 - 592.22
Abortion	1.47	0.00	1.00	6.07	0.24 - 150.92
Intrauterine death	2.94	0.00	0.26	10.26	0.24 - 216.83
	**Children from SARS-CoV-2 positive mothers (n=66)**	**Children from SARS-CoV-2 uninfected mothers (n=136)**	***p-value***	**Odds ratio**	**95% C.I.**
Children weight (g, average ± SD)	2982.27 ± 590.41	3229.46 ± 457.84	*0.004*		
Apgar 1 minute < 7 (%)	6.15	8.09	0.63	0.75	0.23 - 2.44
Apgar 5 minutes < 7 (%)	0.00	1.47	1.00	0.85	0.02 - 8.95
Arterial umbilical pH < 7.0 (%)	23.53	2.21	*0.0000017*	13.64	3.66 - 50.79

SD, Standard Deviation; C.I., Confidence Interval

Table S2. Characteristics of SARS-CoV-2 positive pregnant women and their children associated to the presence or absence of COVID-19 symptoms.

	SARS-CoV-2 positive pregnant women (n=68)				
	**symptomatic (n=24)**	**asymptomatic (n=44)**	***p-value***	**Odds ratio**	**95% C.I.**
Altered non-specific phlogosis indices (%)	75.00	25.58	*0.00012*	8.45	2.67 - 26.75
Preterm birth (%)	22.73	6.98	0.07	3.92	0.84 - 18.29
cesarean section (%)	54.17	29.55	*0.046*	2.82	1.00 - 7.91
operative vaginal birth	0.00	4.55	0.48	0.35	0.02 - 7.53
natural birth	56.62	65.91	0.11	0.44	0.16 - 1.21
Placenta hemorrhagic necrosis (%)	31.25	21.21	0.49	1.69	0.44 - 6.49
Acute intervillositis (%)	0.00	12.12	0.65	0.00	0.00 - 0.00
Placental malperfusion (%)	6.25	9.09	0.73	0.67	0.06 - 6.97
**Chronic diseases (%)**					
total	20.83	15.91	0.74	1.39	0.39 - 4.97
Hypertension	4.17	0.00	1.00	5.68	0.22 - 144.97
Diabetes	8.33	2.27	0.28	3.91	0.34 - 45.52
Hypothyroidism	8.33	6.82	1.00	1.24	0.19 - 8.00
Obesity	4.17	2.27	1.00	1.87	0.11 - 31.29
**Pregnancy-associated complications (%)**					
total	70.83	29.55	*0.00105*	5.79	1.94 - 17.27
Hypertension	8.33	2.27	0.28	3.91	0.34 - 45.52
Diabetes	25.00	13.64	0.32	2.11	0.60 - 7.46
Hypothyroidism	8.33	6.82	1.00	1.24	0.19 - 8.00
Cholestasis	0.00	0.00	0.36	1.82	0.03 - 94.42
Preeclampsia	4.17	2.27	1.00	1.87	0.11 - 31.29
Intrauterine growth restriction	8.33	11.36	1.00	0.71	0.13 - 3.96
Abortion	0.00	2.27	1.00	0.59	0.02 - 15.09
Intrauterine death	8.33	0.00	0.29	9.89	0.46 - 214.83
**Children from SARS-CoV-2 positive mothers (n=66)**					
	**symptomatic (n=22)**	**asymptomatic (n=44)**	***p-value***	**Odds ratio**	**95% C.I.**
Weight (g, average ± SD)	2752.14 ± 528.34	3097.34 ± 586.16	*0.022*		
Apgar 1 minute < 7 (%)	0.00	9.30	0.23	0.20	0.01 - 3.79
Apgar 5 minutes < 7 (%)	0.00	0.00	0.35	1.84	0.04 - 96.12
Arterial umbilical pH < 7.0 (%)	22.22	24.24	0.871	0.89	0.23 - 3.50

SD, Standard Deviation; C.I., Confidence Interval

Table S3. Characteristics of symptomatic SARS-CoV-2 positive mothers and their children as compared to healthy controls.

	**symptomatic SARS-CoV-2 positive women (n=24)**	**SARS-CoV-2 uninfected women (n=136)**	***p-value***	**Odds ratio**	**95% C.I.**
Preterm birth (%)	22.73	9.56	0.07	2.78	0.88 - 8.78
cesarean section (%)	54.17	38.97	0.16	1.85	0.77 - 4.43
operative vaginal birth	0.00	4.41	1.00	0.41	0.02 - 7.51
natural birth	45.83	56.62	0.33	0.65	0.27 - 1.55
**Chronic diseases (%)**					
total	20.83	17.91	0.73	1.21	0.41 - 3.55
Hypertension	4.17	0.00	0.29	17.43	0.69 - 440.71
Diabetes	8.33	0.74	0.06	12.27	1.07 - 141.15
Hypothyroidism	8.33	10.29	1.00	0.79	0.17 - 3.73
Obesity	4.17	0.00	0.29	17.43	0.69 - 440.71
**Pregnancy-associated complications (%)**					
total	70.83	18.38	*0.0000001*	10.78	4.04 - 28.77
Hypertension	8.33	2.21	0.16	4.03	0.64 - 25.51
Diabetes	25.00	5.88	*0.01*	5.33	1.66 - 17.15
Hypothyroidism	8.33	0.00	0.06	30.33	1.41 - 652.78
Cholestasis	0.00	2.21	0.49	0.78	0.04 - 15.55
Preeclampsia	4.17	0.00	0.29	17.43	0.69 - 440.71
Intrauterine growth restriction	8.33	0.00	0.06	30.33	1.41 - 652.78
Abortion	0.00	0.00	0.15	5.57	0.11 - 287.49
Intrauterine death	8.33	0.00	0.06	30.33	1.41 - 652.78
	**children from SARS-CoV-2 symptomatic mothers (n=22)**	**children from SARS-CoV-2 uninfected mothers (n=136)**	***p-value***	**Odds ratio**	**95% C.I.**
Children weight (g, average ± SD)	2752.14 ± 528.34	3229.46 ± 457.84	*0.0006*		
Apgar 1 minute < 7 (%)	0.00	8.09	1.00	0.24	0.01 - 4.26
Apgar 5 minutes < 7 (%)	0.00	1.47	0.37	1.20	0.06 - 25.73
Arterial umbilical pH < 7.0 (%)	22.22	2.21	*0.0038*	12.67	2.57 - 62.43

SD, Standard Deviation; C.I., Confidence Interval

Table S4. Characteristics of asymptomatic SARS-CoV-2 positive mothers and their children as compared to healthy controls.

	**asymptomatic SARS-CoV-2 positive women (N=44)**	**SARS-CoV-2 uninfected women (n=136)**	***p-value***	**Odds ratio**	**95% C.I.**
Preterm birth (%)	**6.98**	**9.56**	**0.605**	**0.71**	**0.19** , 2.62
cesarean section (%)	29.55	38.97	0.26	0.66	0.32 , 1.37
operative vaginal birth	4.55	4.41	1.00	0.96	0.19 , 4.84
natural birth	65.91	56.62	0.28	1.48	0.73 , 3.01
**Chronic diseases (%)**					
total	11.36	17.91	0.31	0.59	0.21 , 1.65
Hypertension	0.00	0.00	0.25	3.07	0.06 , 156.87
Diabetes	2.27	0.74	0.43	3.14	0.19 , 51.27
Hypothyroidism	6.82	10.29	0.77	0.64	0.17 , 2.33
Obesity	2.27	0.00	0.43	9.41	0.38 , 235.33
**Pregnancy-associated complications (%)**					
total	29.55	18.38	0.11	1.86	0.85 , 4.06
Hypertension	2.27	2.21	1.00	1.03	0.10 , 10.17
Diabetes	13.64	5.88	0.11	2.53	0.83 , 7.73
Hypothyroidism	6.82	0.00	0.047	23.02	1.17 , 454.90
Cholestasis	0.00	2.21	1	0.43	0.02 , 8.46
Preeclampsia	2.27	0.00	0.43	9.41	0.38 , 235.33
Intrauterine growth restriction	11.36	0.00	0.004	38.01	2.06 , 702.47
Abortion	2.27	0.00	0.43	9.41	0.38 , 235.33
Intrauterine death	0.00	0.00	0.25	3.07	0.06 , 156.87
	**children from asymptomatic mothers (n=43)**	**children from SARS-CoV-2 uninfected mothers (n=136)**	***p-value***	**Odds ratio**	**95% C.I.**
Children weight (g, average ± SD)	3097.34 ± 586.16	3229.46 ± 457.84	0.181		
Apgar 1 minute < 7 (%)	9.30	8.09	0.76	1.17	0.35 , 3.87
Apgar 5 minutes < 7 (%)	0.00	1.47	0.55	0.65	0.03 , 13.77
Arterial umbilical pH < 7.0 (%)	24.24	2.21	*0.000004*	14.19	3.52 , 57.19

C.I., Confidence Interval; SD, Standard Deviation; Und, undefined
